# A Blockchain Consensus Protocol Based on Quantum Attack Algorithm

**DOI:** 10.1155/2022/1431967

**Published:** 2022-08-22

**Authors:** Hui Wang, Jian Yu

**Affiliations:** ^1^School of Art, Liuzhou Vocational and Technical College, Liuzhou 545006, China; ^2^School of Electronic Information Engineering, Liuzhou Vocational and Technical College, Liuzhou 545006, China

## Abstract

The blockchain is a distributed storage system of digital assets. This decentralized, non-copyable technology stems from universal standard password algorithm and the consensus mechanism of the game theory. The development of quantum computing poses threat to traditional algorithms of blockchain encryption, including symmetric encryption and hash encryption. Focusing on the traditional blockchain consensus mechanism, this paper designs a new blockchain consensus mechanism, based on the stochasticity, irreversibility, and uncertainty of quantum measurement. In the proposed consensus mechanism, complex calculations and intractability mathematical problems are abandoned. In this way, a huge amount of computing resources is saved, less energy is consumed, the time delay is shortened, and the throughput is increased. The proposed quantum consensus mechanism can withstand 51% attacks.

## 1. Introduction

Currently, blockchain has been applied in various fields, such as finance, industry, logistics, Internet of Things (IoT), copyright protection, and data sharing [1–7]. The application scenarios of blockchain provide a high level of data security. To realize decentralization, the blockchain system ensures that consensus tasks are completed without involving third-parties and guarantees the highest level of fault tolerance. The defining features of the system are transparency, credibility, tamper-proofness, forgery prevention, traceability, openness, and fairness [8].

Quantum computers and quantum computing have been developing at a fast speed. Once quantum computers are put into use, the blockchain will be unable to maintain data security. The smart contract system may be hindered, and the entire blockchain technology will go downhill. The security of the blockchain is manifested as the ability to cope with mathematical challenges, which cannot be solved easily even by the most powerful traditional computers.

Quantum passwords have constituted a serious threat to the classic encryption algorithms, such as asymmetric encryption and hash encryption [9, 10]. Shor [11] proposed a quantum algorithm that solves discrete logarithms and integer factors. The algorithm can completely crack Rivest–Shamir–Adleman (RSA) algorithm, digital signature algorithm (DSA), and elliptic curve digital signature algorithm (ECDSA). Grover's algorithm [12] can accelerate traditional hash operations and solve the once unsolvable problems at a reasonable time cost. Hence, the algorithm has attracted much attention in many fields of quantum computing.

## 2. Materials and Methods

Focusing on the consensus mechanism of traditional blockchain, this paper designs a novel blockchain consensus protocol, based on the stochasticity, irreversibility, and uncertainty of quantum measurement. In the proposed consensus mechanism, complex calculations and intractability mathematical problems are abandoned.. In this way, a huge amount of computing resources is saved, less energy is consumed, the time delay is shortened, and the throughput is increased. The proposed quantum consensus mechanism can withstand 51% attacks. The main contributions are as follows.

Firstly, the proposed decentralized consensus mechanism for the blockchain, which is based on quantum encryption technology, achieves higher fault tolerance and attack resistance than traditional mechanisms.

Secondly, quantum entanglement was employed to realize the Byzantine consensus protocol. The entangled state was utilized to construct mutual communication information, which reduces the amount of computing resources consumed in solving hard math problems.

Thirdly, the proposed consensus protocol was tested on a self-designed Ethereum. The test results show that our quantum consensus mechanism can withstand 51% attacks, a sign of its high effectiveness and security.

## 3. Related Work

The consensus protocols based on classic cryptography have been successfully applied in the past two decades [13–15], such as Proof of Work (PoW), Proof of Stake (PoS), Practical Byzantine Fault Tolerance (PBFT), Delegated Proof of Stake (DPoS), Directed Acyclic Graph (DAG)-based algorithm, Paxos consensus algorithm, and raft consensus algorithm, an improvement of Paxos. Based on classic cryptography, these consensus protocols have a high complexity in computing problems like the factorial decomposition of large numbers. Quantum computing threatens the security of these classic protocols, for a huge amount of data can be decomposed in a short time, and the attacker can reconstruct the entire blockchain without being detected. At present, the Byzantine consensus faces serious security threats from network updates and the progress of quantum computing. Based on classic cryptography theories, this consensus mechanism consumes too many resources and energy in the computing process. Many mechanisms are grounded on complex computing, namely, RSA, DSA, ECDSA, elliptic curve (EC) algorithm, ElGamal, and Diffe-Hellman. For instance, RSA2048 can complete cracking within 42 min, using quantum computers [16, 17]. According to the research on traditional cryptography algorithm and quantum computing algorithm, the potential threat of quantum computing to existing cryptography mechanism is shown in Table 1.

To solve the problem, Lyubashevsky proposed a quantum signature scheme [18]and a quantum key distribution strategy [19, 20]thatattempt to to resist quantum computing with quantum technology. Drawing on the unique properties of quantum information, they devised a powerful consensus mechanism based on quantum passwords and integrated quantum encryption protocols into a quantum blockchain. Gottesman and Lu et al. [20–22] put forward a scheme using irreversible one-way functions, which have complex negation operations on a given random input. These one-way functions receive classic bit strings and output quantum states. Behera et al. [23] designed a quantum currency system based on one-way functional security and successfully resisted quantum attacks. Alkadri et al. [24] presented the quantum random prophecy of the quantum random prophecy model and provided a grid-based solution. Harikrishnan et al. [25] proposed the Zero Knowledge Proofs (ZKPs), which verify data without exposing them. Each transaction is assigned to a verifier and a prover. During a ZKP transaction, the verifier tries to prove something to the prover, without disclosing any information about it. To ensure the safety against quantum attacks, two indistinguishable hash functions were integrated to the ZKP protocol. Nevertheless, the above algorithms are not highly scalable, and some of them can only work as auxiliary algorithms. Sun et al. [26] proposed algorithms such as swarm intelligence contract, legal anonymous identity authentication under the consensus protocol.

## 4. Quantum Password-Based Blockchain Consensus Protocol

**Figure 1:**
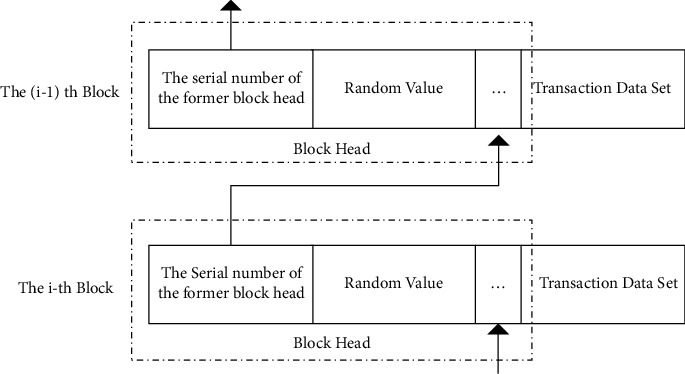
Quantum blockchain.

During the transaction of the quantum blockchain system, miners compete with each other for bookkeeping rights in mining to generate a new blockchain. When users conduct transactions, they need to prepare the quantum state *|ψ* formed by N photon sequences. The network structure of the quantum blockchain is shown in Figure 1. Each block consists of a block head and a block body. The block head contains the sequence number of the previous block head and a random value, and the block body contains a set of transactions.

The traditional consensus protocol distributes keys under the conventional encryption method. If the same ciphertext is sent repeatedly, the attacker Eve will be able to obtain its statistics. This paper designs a consensus protocol, based on the stochasticity, irreversibility, and uncertainty of quantum measurement. Without relying on mathematical proof and complex mathematical calculations, the proposed protocol improves the effectiveness of communication between two parties. Besides, the quantum state as the ciphertext makes it impossible for the attacker to reliably read and analyse the ciphertext. Under the consensus mechanism, the two parties must meet three conditions before communicating with each other: the signatures of the two parties cannot be forged; the signer cannot deny his/her signature; during the communication, any holder of the public key can verify the authenticity of the message.

### 4.1. Quantum Key Distribution Algorithm

To initiate secure communication, the proposed protocol needs to distribute a key and establish a secure channel using that key. If Bob does not have the private code *s*, he will be the owner of non-measurement basis. By random guess alone, he cannot acquire any information about the private code *s* from the certificate sent by Alice. Similarly, if Alice does not have the private code *s*, she will be unable to acquire any information *s* from the authentication process of Bob.

Firstly, it is assumed that Alice does not have the private code *s*, i.e., she does not possess the correct measurement basis. Then, Alice can be regarded as non-honest (or forged by eavesdropper Eve). According to the principle of quantum measurement, unknown quantum states are unclonable. Thus, it is impossible for Eve to obtain the original photon sequence. Then, Eve chooses a random measurement basis for measurement.

According to quantum measurement theory, the quantum state of *N* photon sequences is $|\psi =\left(1/\sqrt{2}\right)\left(\left|0+\right|1\right)$. A random value is generated from [0, 2^*N*^] and encoded as quantum ground state: *|*0⟶0, *|*1⟶1. There is a 50% probability of a random bit that the measurement basis thus chosen is consistent with Alice's measurement basis.

Next, it is assumed that the verifier Bob is forged and does not have the private code *s*. But he wants to steal the private code *s* from the proof process of Alice. Bob chooses a random measurement basis for measurement. The measurement state and measured result *R* can be, respectively, expressed as

(1) $$\begin{array}{c} \left\{|0,|1,|+,|-\right\}. \end{array}$$

The measured result *R* is

(2) $$\begin{array}{c} |0,|+,|0,|-,|1,|+,|1,|-. \end{array}$$

If the measured result *R* is *|*0 or *|*+, Bob will discard the photon sequence and notify Alice to discard the entire photon sequence. If *R* is *|*1or*|*−, Bob will preserve the sequence except 1-bit.

The remaining photons can be encoded as

(3) $$\begin{array}{c} |1>⟶0,|-\;>⟶1. \end{array}$$

Then, Alice chooses a measurement basis to measure the remaining photons. If *R* is *|*0 or *|*+, then Bob is a fake verifier. In this case, Alice will reject the proof and immediately terminate the consensus protocol. If *R* is *|*1or*|*−, the measured result is proved valid. Alice will continue with the following steps.

Alice encodes to obtain her measurement results *S*={*S*_*i*_} according to equation (3) which is the proof information. *S* is encrypted by *K*_*AB*_ and sent to Bob. Bob gets the encrypted proof information from Alice and then decrypts with *K*_*AB*_ and obtains *S*={*S*_*i*_}. If *S*_*i*_=*R*_*i*_, then the verification is passed; else, the above steps will be repeated exchanging the roles of Alice and Bob.

After N iterations, the two particles will hold the same photon sequence and obtain the final shared key. Through the above steps, the two parties can verify each other, completing the interactive authentication under the protocol.

### 4.2. Quantum Consensus Protocol

This section constructs the rules of the consensus protocol with AND operation. Suppose a proposal is waiting to be voted by *n* blockchain nodes (voters) {block_1_,…, block_*n*_}. The vote of each node is represented by a quantum password. Yes and no are denoted by quantum states {*|*0, *|*0} and {*|*1, *|*1}, respectively. Each node can execute quantum AND and quantum OR operations.

The mutual communication of each node is the voting information:

(4) $$\begin{array}{c} v_{i}\in D\left({\mathbb{C}}^{2}\right). \end{array}$$

The voting result of each node can be expressed as

(5) $$\begin{array}{c} v^{j}=\text{AND}\left(v_{1}⊗v_{1}⊗v_{1}⊗,\ldots ,⊗v_{1}\right). \end{array}$$

Each node measures the voting information by the algorithm described in Section 4.1. The measured result is recorded as *R*_1_=|11| and noted as ℝ. For the node set of formula (4), the fault tolerance probability of consensus can be expressed as

(6) $$\begin{array}{c} P\left(v\right)∶=Tr\left(R_{1}v\right). \end{array}$$

That is,

(7) $$\begin{array}{c} Tr^{1}⊗Tr^{2}⊗Tr^{3}R_{i}^{j}\left(|0\rangle \langle 0|⊗\cdots ⊗|0\rangle \langle 0|\right)=Tr\left(|0\rangle \langle 0|\right)\cdot Tr\left(|0\rangle \langle 0|\right)\cdot R_{1}\left(|0\rangle \langle 0|\right)=Tr\left(R_{1}|0\rangle \langle 0|\right)\cdot Tr\left(R_{1}|0\rangle \langle 0|\right). \end{array}$$

Each node has a record set of all quantum voting information. The record set is composed of zeros and ones. For AND(*v*_1_ ⊗ *v*_1_ ⊗ *v*_1_ ⊗ ,…, ⊗*v*_1_), the quantum states may have the same distribution. Then, the quantum voting machine reads all its input and output records. If at least half of the records are 1, then the machine will output “agree”; otherwise, it will output “disagree.”

### 4.3. Security and Performance Analysis

The previous section fully describes the design of our consensus protocol based on quantum encryption. The proposed consensus algorithm utilizes quantum measurement theory, Heisenberg's uncertainty principle, and the quantum no-cloning theorem. The security of the algorithm is independent of the computing power and resources of the computer. Therefore, the algorithm has unconditional security, which is unmatched by classical consensus algorithms.

In practical applications, Alice first encodes the classic photon sequence into the quantum ground state (00101 encoded into |00101) and then applies the quantum gate sequence in accordance with the steps described in Section 4.1. The cost is merely *O*(*n*), a signal of high efficiency. Even if the ciphertext sequence is sent to Bob (or eavesdropped by Eve) via an insecure channel, it is impossible for Eve to duplicate the photon sequence and fake the key, owing to the principle of quantum measurement that unknown quantum states are unclonable. This is because Alice shares the key with Bob via a secure key communication channel, which is based on the quantum sequence. After receiving the ciphertext state, Bob can restore the plaintext through quantum inverse operation. Since the algorithm runs *O*(*n*) times and Alice and Bob exchange information *O*(*n*) times, the protocol needs to send *O*(*n*^2^) messages. The quantum bit consumed by the measurements of each consensus making is a random number *t*. Thus, the mean complexity of our protocol is *O*((*n* − *t*)^2^), where *t* < (*n*/2).

## 5. Experiments and Result Analysis

**Figure 2:**
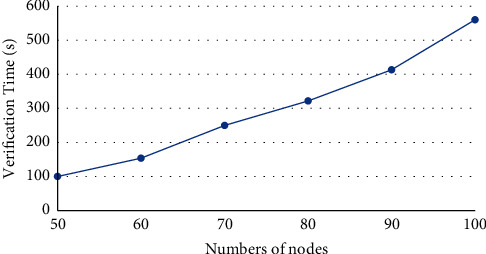
Verification time of security test.

**Figure 3:**
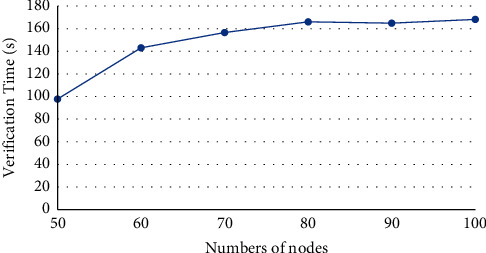
Verification time of fault tolerance test.

**Figure 4:**
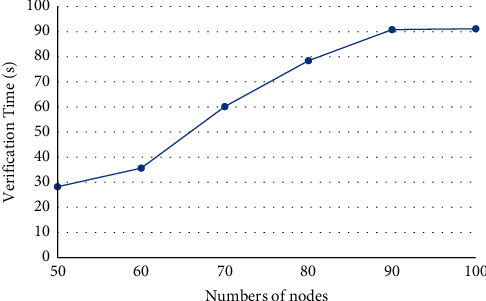
Verification time of mutual communication test.

The software environment of our experiments includes a workstation with Dual-CPU Intel Xeon E5-2440 (2.40 GHz), 64 GB memory, and 64 bit Windows 10 operation system. Based on Ethereum, a private chain was constructed, after deploying geth-windows-amd64-1.8.8 and Ethereum-Wallet-win64-0-11-1. The difficulty was set to 0 × 20000. The experiments mainly test the security, fault tolerance, and mutual communication of our protocol. The security verification means that when all nodes make the same decision, this new block will be allowed to add to the chain and ensure the attacks of selfish-mining or blockchain-fork never happen in the system. The fault tolerance test verifies the resistance of the protocol to forgery attacks: a new block can only be added, if and only if all honest nodes agree with the addition. The mutual communication test verifies if the protocol can withstand 51% attacks. The experimental results (Figures 2–4) demonstrate that our protocol is effective. The effectiveness of the protocol is not greatly affected, with the growing number of nodes.

Our consensus mechanism does not rely on the hash algorithm in the classical blockchain, but the quantum measurement theory and the quantum non-cloning theory, to resist 51% attacks. The traditional blockchain system has the disadvantages of low throughput, long time delay, and high energy consumption. Taking the Bitcoin system as an example, its throughput only allows about 10 transactions per second, and each transaction needs to wait about 10 min to be confirmed. If the transaction is large, the waiting time may reach 1 h. In addition, the system consumes a lot of computing power and energy during the competition of miners. As shown in Figures 2–4, our mechanism can greatly suppress computing power and energy consumption, increase the throughput, and reduce the time delay.

## 6. Conclusions

This paper devises a novel consensus protocol, drawing on the stochasticity, irreversibility, and uncertainty of quantum measurement. Without relying on mathematical proofs and complex mathematical computations, the protocol improves the communication between the two parties of authentication, making the authentication more effective. Since the ciphertext is transmitted in quantum state, the security of ciphertext communication is thus assured, even if the text is eavesdropped multiple times by the attacker. Hence, the consensus mechanism is unconditional and reliable. Compared with the consensus mechanisms based on classic encryption algorithms, the proposed protocol requires relatively few computing resources, consumes only a few energies and a short time, leads to a short time delay, and achieves a high throughput. Experimental results demonstrate that our consensus protocol improves the success rate and effectiveness of the blockchain in resisting quantum attacks and solves a thorny problem of the classic consensus mechanisms: the inability to withstand 51% attacks.

## Figures and Tables

**Table 1:** The potential threats of quantum computation on existing cryptography.

Crypto algorithm	Type	Complex math problem	Security threat

AES	Symmetric	—	In half
SHA256	Hash	—	In half
RSA	Asymmetric	Large integer factoring problem	Complete cracking
ECDSA	Asymmetric	Elliptic curve function	Complete cracking
DSA	Asymmetric	Discrete logarithm	Complete cracking

## Data Availability

The data used to support the findings of this study are available from the corresponding author upon request.
